# Severe Persistent Hyponatremia: A Rare Presentation of Biliary Fluid Loss

**DOI:** 10.1177/2324709619869379

**Published:** 2019-08-17

**Authors:** Asim Kichloo, El-Amir Zain, M. Zatmar Khan, Farah Wani, Jagmeet Singh

**Affiliations:** 1 St. Mary’s Hospital, Saginaw, MI, USA; 2 Central Michigan University, Saginaw, MI, USA; 3 Guthrie Robert Packer Hospital, Sayre, PA, USA

**Keywords:** hyponatremia, acalculous cholecystitis, cholecystostomy, large-volume drainage

## Abstract

Hypotonic hyponatremia is caused by a serum sodium level of <135 mEq/L in the setting of excess solute loss accompanied by free water retention because of antidiuretic hormone release, subsequent to decreased effective arterial blood volume. Acute hyponatremia can have various neurological manifestations, including drowsiness, lethargy, coma, seizures, respiratory depression, and even death. In this article, we present a case of a 41-year-old man who presented with hyponatremia as a result of sodium containing biliary fluid loss and resultant renal free water retention in response to increased antidiuretic hormone secretion. He underwent placement of a cholecystostomy tube for acalculous cholecystitis and was found to be persistently hyponatremic despite repletion with sodium-containing fluids. Once the cholecystostomy tube was removed, the patient’s sodium levels improved, and his symptoms resolved. Our case highlights choleuresis as an unusual but significant cause of hyponatremia in patients who have external biliary drainage.

## Introduction

Hyponatremia is a condition characterized by a serum sodium level of <135 mEq/L.^1^ It is the most common clinical electrolyte imbalance and often occurs in hospitalized patients with a reported incidence in this population of 15% to 30%.^1-3^ Hyponatremia usually presents with neurological symptoms, including lethargy, mental depression, weakness, and confusion,^2^ with the severe symptoms of seizures, obtundation, coma, and respiratory arrest consistent with profoundly low sodium levels.^4^ Unrecognized incidents of hyponatremia are reported to occur for 2 main reasons—lack of screening of fluid and electrolyte levels and misinterpreted changes in serum sodium levels.^5^ The case we present in this article describes severe hyponatremia manifesting with neurological symptoms secondary to biliary losses after external drainage via a cholecystostomy tube for acalculous cholecystitis.

## Case Presentation

A 41-year-old male with past medical history of alcoholic cirrhosis with abstinence from alcohol for 3 years and chronic kidney disease stage 3 presented to the hospital with lethargy and confusion. The patient was on dialysis in the past for a brief period secondary to interstitial nephritis because of Bactrim but with stable kidney functions for the past 3 years. He was last hospitalized 2 months prior for acalculous cholecystitis, which required a cholecystostomy tube. Of note, he was found to have large volume drainage from the cholecystostomy tube. He had drainage volumes of approximately 1.5 to 2 L daily. During the whole time period before his presentation to our facility, he did not report significant symptoms other than the fluid loss mentioned above. Due to the paucity of symptoms, he did not seek medical attention. On subsequent presentation to the hospital, he was administered a liter of normal saline, and laboratory work revealed a serum sodium of 116 mEq/L. His baseline serum sodium prior to these events was around 135 mEq/L, at the time when he had the cholecystostomy drain placement. Furthermore, serum osmolality was found to be 258 mOsm/kg, urine osmolality of 111 mOsm/kg, and a low urine sodium excretion measuring <20 mEq/L. The laboratory results were suggestive of low solute or hypotonic hyponatremia. His biliary fluid spot sodium was found to be 154 mEq/L.

The patient was initially managed with intravenous normal saline followed by oral sodium chloride supplementation (2 g twice daily) and free water restriction of <1 L per day. Despite these measures, his serum sodium remained low and was typically between 120 and 130 mEq/L. At this point, a decision was made to clamp the cholecystostomy tube and subsequently remove it. This resulted in a gradual improvement of serum sodium with a rise in serum sodium levels to more than 135 mEq/L. The rise in serum sodium was consistent with the improvement in neurological symptoms and lethargy. The patient was later discharged with no further sodium supplementation.

## Discussion

Most cases of hyponatremia arise due to an inability to excrete free water relative to the body’s sodium. One of the subsets of hyponatremia is hypovolemic hyponatremia, which means that a patient is volume-depleted with high sodium losses accompanying fluid loss. Severe hypotonic hyponatremia in adults is often caused by use of thiazide diuretics, syndrome of inappropriate antidiuretic hormone secretion, and a postoperative state (which is often due to administration of hypotonic fluids regardless of the type of procedure).^2,6^ Severe hyponatremia can cause lethargy, coma, and seizures. It may progress to respiratory depression and death if it is not treated appropriately.^2^

One unusual cause of hypotonic hyponatremia is biliary drainage.^4,7^ Sodium concentration in the bile is usually between 122 and 164 mEq/L with the average falling around 145 mEq/L.^4^ This patient had about 2 L of bile drainage daily, which accounted for about 290 mEq per day. This meant that the patient was losing around 6 or 7 g of sodium each day. This amount was more than the amount that was administered in attempted treatments, resulting in persistent severe hyponatremia. Patients like our case who have external biliary drainage and hyponatremia may have hyponatremia caused by large volume losses of bile. Thus, such patients need close monitoring of electrolyte status and prompt correction of electrolyte abnormalities to avoid adverse effects. Risk factors for the development of hyponatremia in patients include smaller volumes of bile loss over longer periods of time, preexisting renal disease, adrenocortical insufficiency, restriction of sodium in patients’ diets, and diuretic treatment.^4^ This patient also had a history of alcoholic cirrhosis. Even mild reductions in sodium levels puts patients with cirrhosis at a high risk of having severe ascites and the complications associated with it.^8^ Cirrhosis is reported to cause hyponatremia by means of neurohormonal activation that directly correlates with the severity of the hyponatremia.^3^ This patient’s history of alcoholic cirrhosis likely also increased his risk of developing hyponatremia.

Also of importance in this case report is the use of a cholecystostomy tube. Cholecystostomy tubes have been used as a means of drainage in patients, such as after laparoscopic hepatic exploration to prevent the leakage of bile and patients with cholecystitis who are high risk for surgery because of increased mortality like the patient reported in this case report. Reported side effects of such tubes include bile loss, infection at the site of the tube, and displacement of the tube.^9^ It has been reported that tube drainage after common duct exploration is associated with high-volume biliary output.^6^ This patient had excessive bile loss through their tube, and clamping of the tube paired with treatment administration allowed for resolution of the patient’s hyponatremia. Patients with such tubes should have their sodium levels checked to ensure that severe electrolyte imbalances are avoided or appropriately handled.

Therapy with isotonic fluids containing sodium, chloride, lactate, bicarbonate, and potassium in patients with hyponatremia should be based on measurements of biliary fluid volume and electrolyte concentrations. In patients with cholecystostomy tubes, biliary fluid loss can also be avoided by conversion to internal biliary drainage as opposed to external biliary drainage in some obstructive conditions. The benefits of internal drainage include more ways to access the biliary tree and the lack of an external catheter, which improves the patient’s quality of life.^10^ It also has been shown that internal drainage is preferred after certain procedures, such as hepatectomy, in which external drainage suppresses liver regeneration in certain conditions but internal drainage does not.^11,12^ Other differences between external and internal drainage includes the potential for contamination, which only considered a risk for external tubes, length of the drainage systems, and impedance to flow, which is lower with internal drainage.^13^

## Conclusion

External biliary drainage may cause severe persistent hyponatremia in patients, which may present with an array of symptoms. Although a rare cause of hypovolemic hyponatremia, it may occur due to excessive loss of bile fluid. Prompt diagnosis allows for faster treatment and the prevention of irreversible damage caused by the electrolyte imbalance. Patients with cirrhosis, large losses of bile, and previous renal failure are particularly of note due to the increased incidence of hyponatremia in these patients. All these factors put this patient at a higher risk of the development of hyponatremia in conjunction with bile loss via cholecystostomy tubes. Clinicians should be mindful of the risk factors and signs of hyponatremia in this patient population.

## References

[1] Verbalis JG Disorders of water balance. In: K Skorecki GM Chertow PA Marsden MW Taal ASL Yu, eds. Brenner and Rector’s the Kidney. 10th ed. Philadelphia, PA: Elsevier; 2016:460-510.

[2] Adrogué HJ Madias NE. Hyponatremia. N Engl J Med. 2000;342:1581-1589. 10824078 10.1056/NEJM200005253422107

[3] Upadhyay A Jaber BL Madias NE. Incidence and prevalence of hyponatremia. Am J Med. 2006;119(7 suppl 1):S30-S35. 10.1016/j.amjmed.2006.05.005 16843082

[4] Furey AT. Hyponatremia after choledochostomy and T tube drainage. Am J Surg. 1966;112:850-855. 5923802 10.1016/0002-9610(66)90137-1

[5] Basten A Opit LJ. Severe sodium depletion with renal failure following common bile duct drainage. Aust N Z J Surg. 1966;36:105-110. 5224803

[6] Chung HM Kluge R Schrier RW Anderson RJ. Postoperative hyponatremia. A prospective study. Arch Intern Med. 1986;146:333-336. 3947194

[7] Sandborn WJ Gross JB Jr Larson DE Phillips JK Lindor KD. High-volume postobstructive choleresis after transhepatic external biliary drainage resolves with conversion to internal drainage. J Clin Gastroenterol. 1993;17:42-45. 8409297 10.1097/00004836-199307000-00012

[8] Angeli P Wong F Watson H Ginès P; CAPPS Investigators. Hyponatremia in cirrhosis: results of a patient population survey. Hepatology. 2006;44:1535-1542. 17133458 10.1002/hep.21412

[9] Zeng X Yang P Wang W. Biliary tract exploration through a common bile duct incision or left hepatic duct stump in laparoscopic let hemihepatectomy for left side hepatolithiasis: which is better? A single-center retrospective case-control study. Medicine. 2018;97:e13080. 10.1097/MD.0000000000013080 PMC6257484 30431577

[10] Nussbaum JS Kumta NA. Endoscopic ultrasound-guided biliary drainage. Gastrointest Endosc Clin N Am. 2019;29:277-291. 30846153 10.1016/j.giec.2018.11.005

[11] Suzuki H Iyomasa S Nimura Y Yoshida S. Internal biliary drainage, unlike external drainage, does not suppress the regeneration of cholestatic rat liver after partial hepatectomy. Hepatology. 1994;20:1318-1322. 7927267

[12] Saiki S Chijiiwa K Komura M Yamaguchi K Kuroki S Tanaka M. Preoperative internal biliary drainage is superior to external biliary drainage in liver regeneration and function after hepatectomy in obstructive jaundiced rats. Ann Surg. 1999;230:655-662. 10561089 10.1097/00000658-199911000-00007 PMC1420919

[13] Lygidakis NJ. Acute suppurative cholangitis: comparison of internal and external biliary drainage. Am J Surg. 1982;143:304-306. 7065350 10.1016/0002-9610(82)90096-4

